# Natural Compound Library Screening Identifies Oroxin A for the Treatment of Myocardial Ischemia/Reperfusion Injury

**DOI:** 10.3389/fphar.2022.894899

**Published:** 2022-05-12

**Authors:** Xingdong Cheng, Tingting Huang, Chunhui Wang, Shuang Hao, Liliang Shu, Shixiong Wang, Gao Cheng, Qiaoyun Zhang, Jian Huang, Chen Chen

**Affiliations:** ^1^ Department of Anesthesiology, The Forth Affiliated Hospital of Anhui Medical University, Hefei, China; ^2^ Second Clinical Medical College, Lanzhou University, Lanzhou, China; ^3^ Department of Cardiovascular Surgery, The First Affiliated Hospital of Zhengzhou University, Zhengzhou, China

**Keywords:** high-throughput drug screening, myocardial ischemia/reperfusion injury, oxygen-glucose deprivation/reperfusion, oroxin A, pyroptosis

## Abstract

Myocardial ischemia/reperfusion injury (MI/RI) is a serious pathophysiological process relating to cardiovascular disease. Oroxin A (OA) is a natural flavonoid glycoside with various biological activities. However, its effect on the pathophysiological process of MI/RI has not yet been reported. The aim of this study was to determine whether OA could alleviate MI/RI induced inflammation and pyroptosis *in vivo* and *in vitro*, providing a novel therapeutic regimen for the treatment of MI/RI. A high-throughput drug screening strategy was employed to test 2,661 natural compound libraries that can alleviate MI/RI *in vivo* and *in vitro*. The rat model of MI/RI was established by ligating the left anterior descending (LAD) coronary artery. H9c2 cells were subjected to oxygen-glucose deprivation/reperfusion (OGD/R) to simulate MI/RI. The results show that OA is able to significantly inhibit apoptosis, pyroptosis and the inflammation response (TNF-α, IL-6, IL-8, IL-10, IL-1β, IL-18) *in vivo* and *in vitro*, and reduce the release of myocardial enzymes (cTnI, cTnT, CK-MB, LDH, AST). In the rat MI/RI model, OA can not only improve cardiac function and reduce inflammatory cell infiltration but also reduce myocardial infarct size. The results revealed that OA is an effective remedy against MI/RI as it reduces the inflammatory response and inhibits pyroptosis. This may provide a new therapeutic target for the clinical treatment of MI/RI.

## Introduction

Acute myocardial infarction (AMI) refers to a decrease in blood flow to the coronary arteries, resulting in insufficient oxygen supply and nutrients to the heart (Milanlouei et al., 2020). Currently, reperfusion therapy is the standard treatment for AMI (Shen et al., 2022). However, reperfusion often leads to inflammation and myocardial ischemia/reperfusion injury (MI/RI), which greatly influences morbidity and mortality following AMI (Shen et al., 2022). The pathogenesis of MI/RI is an extremely complex process, which may lead to myocardial systolic dysfunction, reperfusion arrhythmia, myocardial shock, and fatal reperfusion injury (Milanlouei et al., 2020; Seefeldt et al., 2021). Therefore, obtaining a safe and effective drug treatment for MI/RI is paramount and exploring its mechanism of action is still the focus of current and future research.

A growing body of evidence suggests that inflammation and cardiomyocyte pyroptosis play important roles in MI/RI progression (Shi et al., 2021; Ou et al., 2022; Shen et al., 2022). The inflammatory response may be the triggering factor of AMI, and the expression of many inflammatory factors are visible in the pathophysiological process of MI/RI. Pyroptosis, a recently identified type of programmed cell death, is implicated in the inflammatory cascade (Yu et al., 2021). It is dependent on the activation of the NOD-like receptor protein 3 (NLRP3) inflammasome, which utilizes the apoptosis-associated speck-like protein (ASC) to recruit and activate Caspase-1 (Gu et al., 2022).

High-throughput screening (HTS) has become a fundamental tool in modern drug discovery that is often used to uncover new, effective treatments for intractable diseases (Moya et al., 2021). In this study, a HTS strategy was employed to discover new drugs that could mitigate MI/RI. A library of 2,661 natural product compounds was screened when performing oxygen-glucose deprivation/reperfusion (OGD/R) on rat embryonic cardiomyocyte (H9c2) cells. A specific emphasis is placed on Oroxin A (OA), as it was observed to significantly resist OGD/R damage to H9c2 cells.

OA is also known as baicalein-7-O-glucoside, which is an active flavonoid component isolated from the traditional Chinese medicinal herb *Oroxylum indicum (L.) Kurz* (OILK) (earlier name: *Oroxylum flavum Rehder*; Bignoniaceae) (Sun et al., 2018; Pei et al., 2022). OILK has been used safely for centuries in China, India and other Southeast Asian countries (Dinda et al., 2015). It has been widely used in the treatment of gastrointestinal diseases and respiratory diseases (Dinda et al., 2015; Rai et al., 2021). However, the effect of OA on MI/RI has not yet been reported.

In this study, the protective effect of OA on MI/RI through *in vivo* and *in vitro* experiments is investigated and explores the underlying mechanism by which OA protects against MI/RI.

## Material and Methods

### Establishment of H9c2 Cells for OGD/R Model

H9c2 cells were obtained from the Cell Bank of the Chinese Academy of Sciences. The cells were cultured in Dulbecco’s Modified Eagle’s Medium (DMEM, Gibco, United States), supplemented with 10% fetal bovine serum (FBS, Gibco, United States) and 1% penicillin/streptomycin (Solarbio, China), and grown in a humidified incubator containing 5% CO_2_ and 95% air at 37°C. The OGD/R model was used to mimic MI/RI conditions *in vitro*. The culture medium was replaced with glucose-free DMEM (Solarbio, China) without FBS and the cells were cultured in a three-air incubator containing 1% O_2_ and 5% CO_2_ (Thermo Fisher, United States) at 37°C for 12 h. After this time, the cells were incubated with DMEM (containing 10% FBS) in normoxic conditions (95% air, 5% CO_2_) at 37°C for 12 h as a reperfusion process. The cell morphology and growth status were observed under a light microscope (Nikon, Japan).

### High-Throughput Natural Product Library Screenings and Cell Grouping

Natural Product Library and OA were purchased from Selleck (Houston, United States). H9c2 cells (10^4^ cells per well) were seeded on 96-well plates and cultured in the incubator (95% air, 5% CO_2_) at 37°C for 24 h. Subsequently, 2,661 natural compounds were added into each well at a final concentration of 10 μM at the initiation of OGD/R. At the end of the experiment, the cell viability was detected by Cell Counting Kit-8 (CCK-8, Biosharp, China) according to the manufacturer’s instructions and the screened candidate compound, which was OA. To determine the optimal concentration of OA administration, H9c2 cells were treated by different concentrations, which were 5 μM, 10 μM, 100 μM, and 1,000 μM, respectively. Finally, 10 μM was used as the optimal concentration for subsequent experiments. The cells were randomly assigned either into the Control group, the OGD/R group, or the OGD/R + OA group. The latter group represents those cells that were treated with 10 µM OA and subjected to OGD/R procedure.

### Ethical Approval and Establishment of MI/RI Rat Model

**FIGURE 1:**
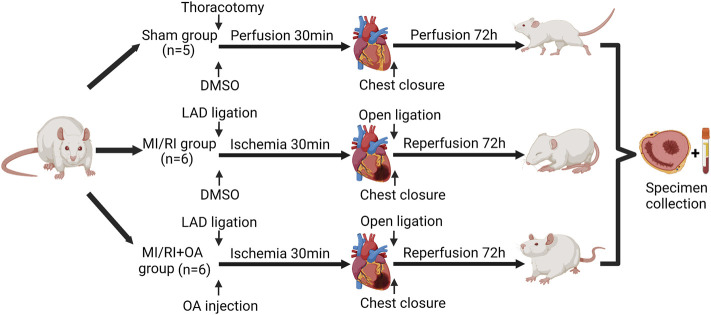
Flow chart of the experimental protocol. We acknowledge the use of Biorender to generate Figure 1.

The study protocol was approved by Laboratory Animal Ethics Committee of Anhui Medical University [LISC 20180351] and performed according to the guidelines of the US Department of Health for the Use and Care of Laboratory Animals. The animals were housed in a temperature-controlled room with a 12-h light–dark cycle and free access to water and food. Seventeen male Sprague-Dawley rats (320 ± 50 g) were used in the study. Rats were randomly divided into three groups: Sham group (n = 5), MI/RI group (n = 6) and MI/RI + OA group (n = 6). Figure 1 illustrates the flow chart of the experimental operation. Anesthesia was induced using 5% sevoflurane. Following was an endotracheal intubation in the supine position using a 16-gauge cannula mechanically ventilated under a respiratory frequency of 70–75 breaths/min, a tidal volume of 6 ml/kg, and an I:E ratio of 1:2 using a mini-ventilator (Alcott Biotech, China). Stable anesthesia was maintained using 1.5% sevoflurane throughout the procedure. OA was dissolved in dimethylsulfoxide (DMSO, Sigma, United States) and stored at 4 °C. Rats in the MI/RI + OA group were administered 5 mg/kg OA by tail intravenous injection at the onset of ischemia. The same doses of DMSO were provided to both the Sham and MI/RI groups. After the operating area was shaved and sterilized, the thorax was opened at the fourth intercostal space. For both the MI/RI group and the MI/RI + OA group, a 5–0 prolene suture was applied to induce ischemia by ligating the left anterior descending (LAD) coronary artery for 30 min. After 30 min of ischemia, the ligation was opened to allow for reperfusion for 72 h. The Sham group underwent the same surgical procedures without blood occlusion of the LAD. All rats received carprofen (5 mg/kg) for postoperative analgesia. After 72 h of reperfusion, rats were euthanized using barbiturate overdoses (pentobarbital) administered intravenously. Subsequently, cardiac tissue and blood samples were collected for further evaluation.

### Cell Viability Assay

At the end of the experiment, the CCK-8 reagent was added to each well and incubated (95% air, 5% CO_2_) for one hour at 37°C. The optical density values at 450 nm were measured using a microplate reader (Thermo Fisher, United States). The ratio of cell viability was calculated using the following formula: Cell viability (%) =(V_experiment_ − V_blank_)/(V_control_ − V_blank_)×100%.

### Cell Apoptosis Analysis

The cells from each group were seeded in 6-well plates. The cell apoptosis was examined using Hoechst 33,258 (Beyotime, China) staining per the instructions from the manufacturer. Finally, the nuclear morphology of each group of cells was observed under a fluorescence microscope (Olympus, Japan).

### Western Blot Analysis

Total proteins from the H9c2 cells and myocardial tissues were extracted using radioimmunoprecipitation (RIPA) lysis buffer (Beyotime, China) containing protease inhibitor. Proteins were quantified using bicinchoninic acid assay (BCA, Solarbio, China). Equal amounts of proteins from each sample were separated by sodium dodecyl sulfate-polyacrylamide gel electrophoresis and transferred to polyvinylidene difluoride (PVDF) membranes. Subsequently, the membranes were blocked with 5% non-fat milk for 1 h at room temperature and incubated with primary antibodies anti-NLRP3 (1:2000, Abcam, United States), anti-Caspase1 (1:4000, Proteintech, China) and anti-GSDMD (1:4000, Proteintech, China) overnight at 4 °C, β-actin (1:5000, Proteintech, China) was used as reference gene in the experiment. PVDF membranes were incubated with appropriate horseradish peroxidase conjugated secondary antibodies for 1 h at 37 °C. Finally, immunoreactivity was detected with enhanced chemiluminescence detection reagents (Millipore, United States) in the ChemiDoc MP Gel Imaging System (Bio-Rad, United States).

### Reverse-Transcription Quantitative PCR (RT-qPCR) Assay

Total RNA was isolated from the H9c2 cells and myocardial tissues using Trizol^®^ reagent (Takara, Japan). The RNA yield was determined by a NanoDrop™ spectrophotometer (Thermo Fisher, United States). Total RNA from the sample was reverse transcribed to complementary DNA using a commercially available kit according to the manufacturer’s instructions. RT-qPCR was performed using the CFX96TM Real-time Detection System (Bio-Rad, United States) and TB Green qPCR Mix Plus (Takara, Japan). A quantification of the mRNA levels was performed using the delta cycle time method. All samples and controls are normalizations against *β-actin*, which is the reference gene. The rat gene-specific primer sequences are provided in Table 1.

**TABLE 1:** RT-qPCR primers

**Target gene**	**Primer sequence**
*nlrp3*	Forward: 5ʹ-GTGTGGATCTTTGCTGCGAT-3ʹ
	Reverse: 5ʹACAAATGGAGATGCGGGAGA-3ʹ
*caspase1*	Forward: 5ʹ-ACAAGGCACGGGACCTATG-3ʹ
	Reverse:5ʹ-TCCCAGTCAGTCCTGGAAATG-3ʹ
*gsdmd*	Forward: 5ʹ-CCAGCATGGAAGCCTTAGAG-3ʹ
	Reverse: 5ʹ-CAGAGTCGAGCACCAGACAC-3ʹ
*β-actin*	Forward: 5-CACCATTGGCAATGAGCGGTTC−3’
	Reverse: 5-AGGTCTTTGCGGATGTCCACGT−3’

*nlrp3*: NOD-like receptor protein 3; *gsdmd*: gasdermin D.

### Histopathological Analysis

The myocardial tissues were excised and fixed with 4% paraformaldehyde. All tissues were embedded in paraffin and cut into 5 μm thick sections. Hematoxylin and eosin (H&E) (Beyotime, China), immunohistochemistry (IHC) and terminal-deoxynucleotidyl transferase-mediated nick end labeling (TUNEL) staining were performed according to the instructions from the manufacturer. For the H&E staining, the heart injury score was described as follows: 0 = no injury, 1 = isolated myocardial injury, 2 = an area of injury, 3 = two or more areas of injury, and 4 = over 50% of the areas of diffuse myocardial injury (Seo et al., 2018). For the IHC staining, the sections were incubated at 4°C overnight with the primary antibodies anti-Myeloperoxidase (anti-MPO) (1:2000, Abcam, United States) and anti-CD68 (1:1,000, Abcam, United States). Visualization was achieved with peroxidase-labeled streptavidin-biotin and diaminobenzidine staining. A histological quantification and analysis were performed in a double-blinded manner.

### Enzyme-Linked Immunosorbent Assay

The levels of cTnI, cTnT, CK-MB, LDH, AST, TNF-α, IL-6, IL-8, IL-10, IL-1β and IL-18 in the serum, heart tissues and cell culture medium were measured using ELISA assay (enzyme-linked Biotech, China) per the manufacturer’s instructions.

### 2,3,5-Triphenyltetrazolium Chloride (TTC) Staining

At the end of the experiment, the hearts of the rats were removed and rapidly frozen at -20 °C for 15 min. Subsequently, the frozen hearts were each cut into five slices and incubated at 37°C in 2% TTC solution (Solarbio, China) for 18 min. Thereafter, the sections were fixed with 4% paraformaldehyde and photographed. The proportion of the infarcted area (white) was analyzed using ImageJ 8.4 software (National Institutes of Health, United States).

### Echocardiography

At the end of 72-h reperfusion period, the rats were anaesthetized with 2% isoflurane. The cardiac function was evaluated using M-mode echocardiography with a 22 MHz linear transducer (EPIQ7, Philips, Netherlands). The ejection fraction (EF), fractional shortening (FS), end-diastolic volume (EDV) and end-systolic volume (ESV) were measured using Vevo LAB 3.1.0 software.

### Statistical Analysis

All of the measured and calculated values were expressed as the mean ± standard deviation (SD). Data from two independent groups were compared using the Mann–Whitney *U* test. GraphPad Prism software (Version 9.2.0; GraphPad Software Inc., San Diego, California, United States) was used for statistical analysis. All *p*-values were adjusted with Turkey’s test for multiple comparisons, where *p*-values < 0.05 were considered as statistically significant.

## Results

### HTS of Natural Product Library Identified Compounds Against OGD/R Induced H9c2 Cells Injury

**FIGURE 2:**
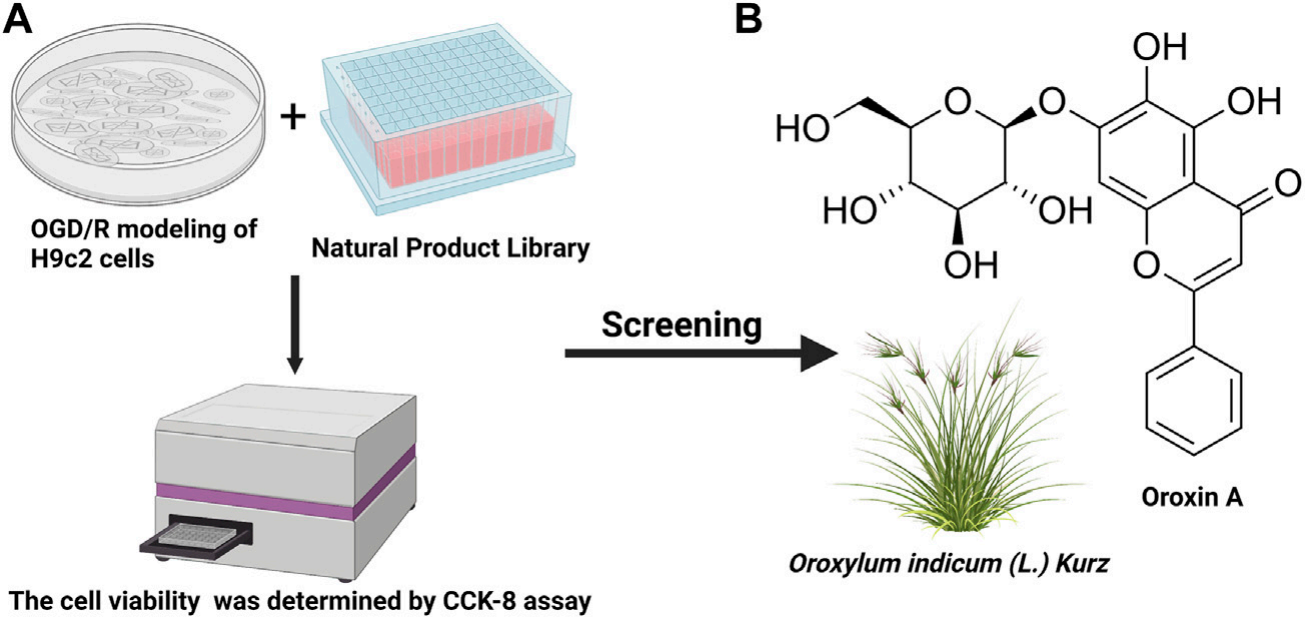
HTS was used to determine the most promising compound **(A)** The schematic diagram of the drug screening protocol **(B)** Chemical structure of OA. HTS: High-throughput screening; OGD/R: oxygen-glucose deprivation/reperfusion; CCK-8: cell counting kit-8; OA: Oroxin A. We acknowledge the use of Biorender to generate Figure 2.

To identify a drug that could protect H9c2 cells from ischemia-reperfusion injury, a HTS strategy was used to select a drug candidate from a library of 2,661 natural compounds. The schematic diagram of the drug screening protocol was shown in (Figure 2A). The potential effects of compounds on the viability of OGD/R treated H9c2 cells were evaluated using CCK-8 assay. An appropriate candidate compound, known as OA, was identified for this study (Figure 2B).

### OA Treatment Attenuates OGD/R Induced H9c2 Cells Injury

**FIGURE 3:**
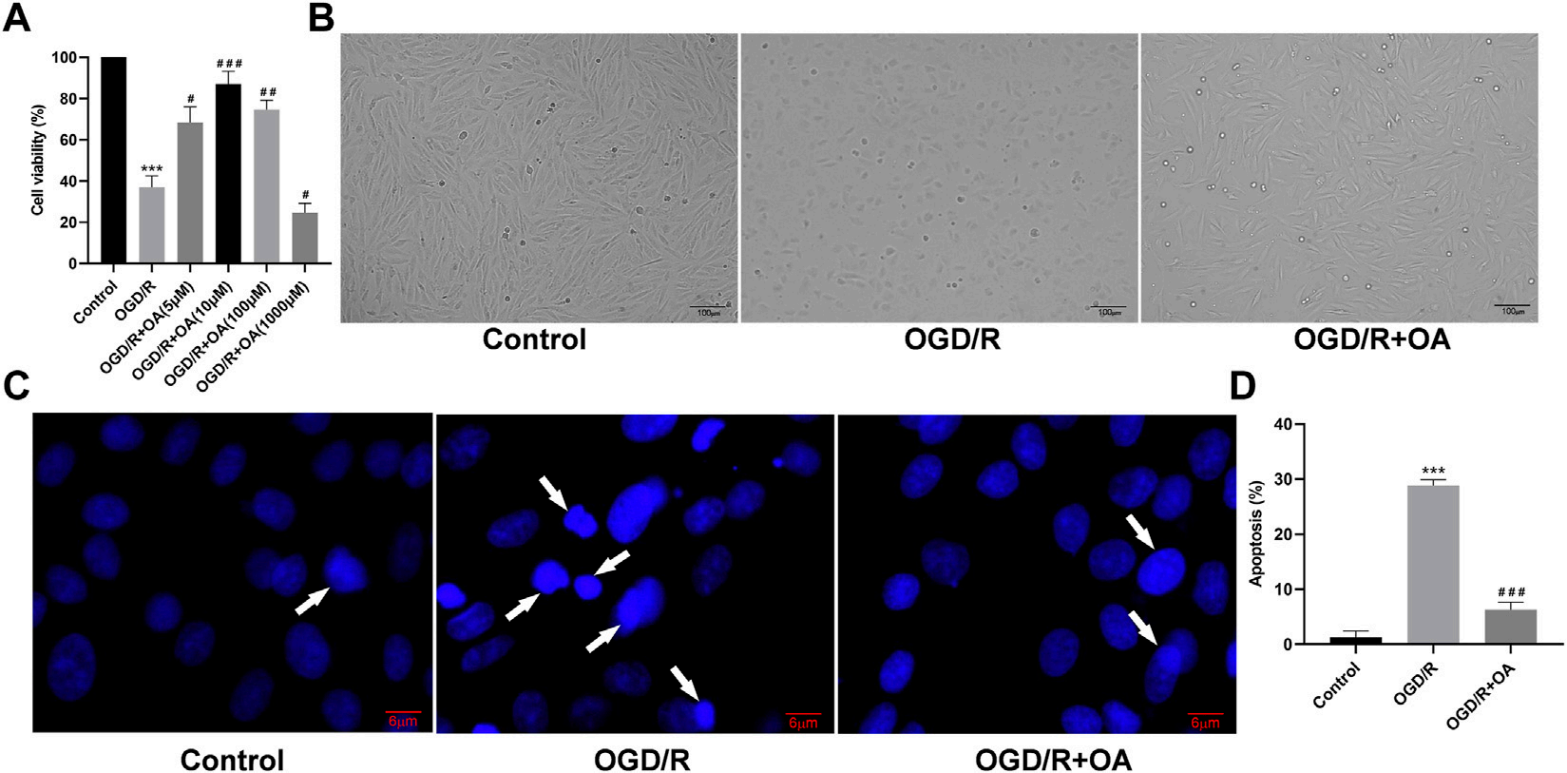
OA treatment attenuates OGD/R induced H9c2 cells injury **(A)** Cell viability of the OGD/R + OA group was the highest compared with the OGD/R group at a dose of 10 μM. When the concentration was higher than 10 μM, the cell viability gradually decreased **(B)** Representative images of H9c2 cells morphological changes in each group (scale bar = 100 μm) **(C,D)** Representative Hoechst 33,258 images and quantitative analysis of apoptotic ratio for H9c2 cells in each group (scale bar = 6 μm). Arrows indicate representative apoptotic positive cells. Data are presented as the mean ± SD. ^∗∗∗^ *p* < 0.001, compared with the Control group; ^#^ *p* < 0.05, ^##^ *p* < 0.01, and ^###^ *p* < 0.001, compared with the OGD/R group. OGD/R: oxygen-glucose deprivation/reperfusion; OA: Oroxin A.

To confirm optimal intervention concentration of the OA in the H9c2 cells for the OGD/R model, the cell viability was explored using CCK-8 assay. The results show that the viability of OGD/R-treated H9c2 cells are significantly lower when compared with the Control group (100.00 ± 5.57 *vs* 37.00 ± 5.51, *p* < 0.001; Figure 3A). However, OA treatment markedly increased the cell viability after OGD/R injury. The cell viability of the OGD/R + OA group was the highest compared with that of the OGD/R group at a dose of 10 µM (37.00 ± 5.51 *vs* 87.00 ± 6.25, *p* < 0.001; Figure 3A). When the concentration was higher than 10 μM, the cell viability gradually decreased. Thus, 10 μM OA were utilized for the subsequent experiments. The cell morphology was detected by a light microscope. The morphology of the cells that were exposed to OGD/R changed from a spindle shape to a round shape. The cells contracted, and the refractive index decreased significantly. However, OA intervention significantly stabilize cell morphology (Figure 3B). Additionally, the effects of OA on OGD/R induced apoptosis were measured using Hoechst 33,258. Figure 3C displayed many bright blue nuclear condensation and fragmentation following OGD/R treatment. The percentage of apoptotic cells in the OGD/R group was markedly higher than that in the Control group (1.23 ± 1.17% *vs* 28.83 ± 1.04%, *p* < 0.001; Figure 3D). Conversely, OA treatment reduced the apoptosis rate.

### OA Treatment Attenuates the Expressions of Pyroptosis Related Proteins and mRNA After OGD/R

**FIGURE 4:**
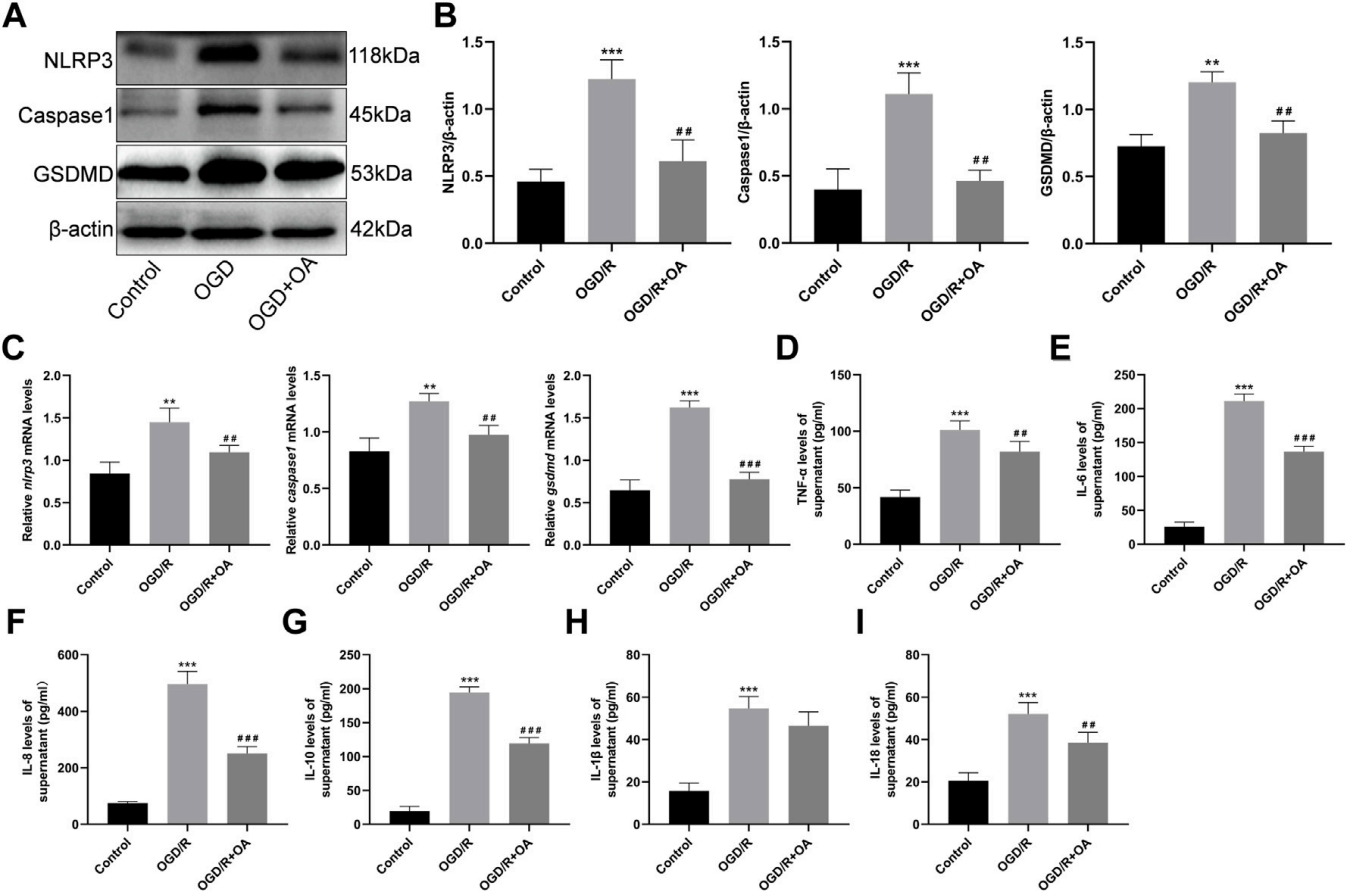
OA treatment attenuates OGD/R induced pyroptosis and inflammation **(A,B)** WB and quantitative analysis for NLRP3, Caspase1, and GSDMD expression levels in the heart tissues **(C)** RT-qPCR data showing the transcripts levels for *nlrp3, caspase1, and gsdmd* in the heart tissues **(D–I)** ELISA results show that the levels of TNF-α, IL-6, IL-8, IL-10, IL-1β and IL-18 in the supernatant in each group. Data are presented as the mean ± SD. ^∗∗^ *p* < 0.01 and ^∗∗∗^ *p* < 0.001, compared with the Control group; ^##^ *p* < 0.01 and ^###^ *p* < 0.001, compared with the OGD/R group. OGD/R: oxygen-glucose deprivation/reperfusion; OA: Oroxin A; WB: Western Blot; RT-qPCR reverse-transcription quantitative PCR; ELISA: enzyme-linked immunosorbent assay.

The expressions of pyroptosis related proteins were detected by WB (Figure 4A). Compared with the Control group, the NLRP3, Caspase1, and GSDMD protein expressions were significantly higher in the OGD/R group (0.46 ± 0.09 *vs* 1.22 ± 1.14, *p* < 0.001, 0.40 ± 0.15 *vs* 1.11 ± 0.16, *p* < 0.001, and 0.73 ± 0.09 *vs* 1.20 ± 0.08, *p* < 0.01, respectively; Figure 4B). However, OA treatment markedly decreased the NLRP3, Caspase1, and GSDMD protein expressions following OGD/R. The RT-qPCR results show that the levels of *nlrp3*, *caspase1*, and *gsdmd* mRNA are significantly higher in the OGD/R group when compared with the Control group (0.84 ± 0.13 *vs* 1.45 ± 0.17, *p* < 0.01, 0.83 ± 0.12 *vs* 1.27 ± 0.07, *p* < 0.01, and 0.65 ± 0.12 *vs* 1.62 ± 0.08, *p* < 0.001; Figure 4C). However, OA treatment markedly decreased the levels of *nlrp3*, *caspase1*, and *gsdmd* mRNA after OGD/R.

### OA Treatment Attenuates the Inflammatory Cytokines Levels of Cell Supernatants

To determine whether OA could attenuate the inflammatory cytokines levels of cell supernatants, an ELISA analysis was conducted. The results show that the levels of TNF-α, IL-6, IL-8, IL-10, IL-1β and IL-18 are significantly higher in the OGD/R group when compared with the Control group (41.84 ± 6.05 *vs* 101.20 ± 7.99, 26.02 ± 6.56 *vs* 211.10 ± 10.39, 75.46 ± 4.68 *vs* 496.40 ± 44.68, 19.18 ± 7.14 *vs* 194.40 ± 8.46, 15.70 ± 3.79 *vs* 54.67 ± 5.67, and 20.54 ± 3.84 *vs* 52.10 ± 5.39, *p* < 0.001; Figure 4D–I). Conversely, the levels of the inflammatory cytokines in the OGD/R + OA group were markedly reduced when compared to that in the OGD/R group.

### OA Treatment Ameliorates MI/RI *in vivo*

**FIGURE 5:**
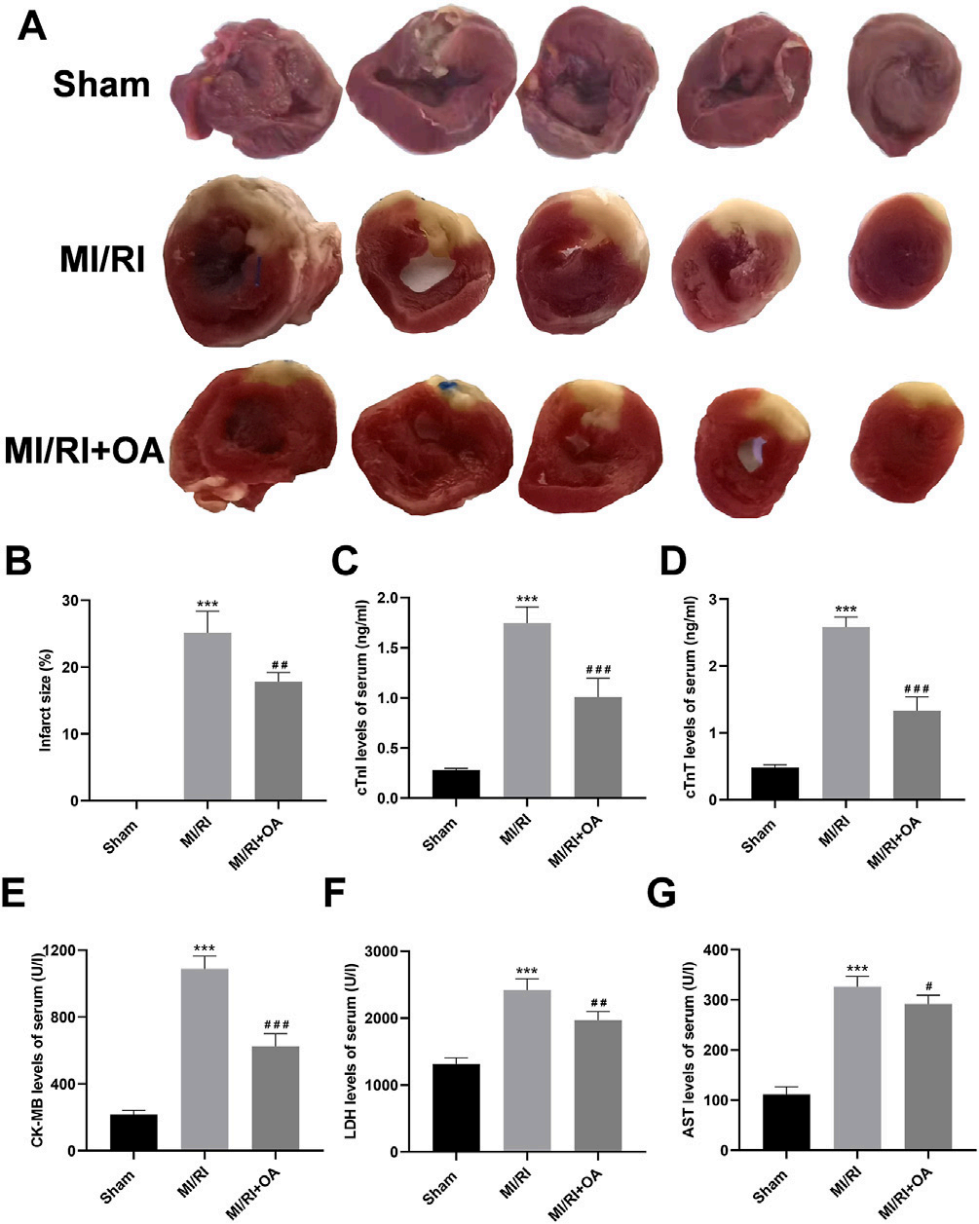
OA treatment ameliorates MI/RI *in vivo* **(A,B)** Representative TTC staining images and quantitative analysis for the heart tissues in each group **(C–G)** ELISA results showing that serum levels of cTnI, cTnT, CK-MB, LDH, AST in each group. Data are presented as the mean ± SD. ^∗∗∗^ *p* < 0.001, compared with the Sham group; ^#^ *p* < 0.05, ^##^ *p* < 0.01, and ^###^ *p* < 0.001, compared with the MI/RI group. MI/RI: myocardial ischemia/reperfusion injury; OA: Oroxin A; TTC: 2,3,5-Triphenyltetrazolium chloride; ELISA: enzyme-linked immunosorbent assay.

To further confirm the role of OA *in vivo*, we established a rat model of MI/RI. Myocardial infarct size was detected by TTC staining (Figure 5A). The results show that the infarct size are significantly larger in the MI/RI group when compared with the Sham group (0.02 ± 0.01 *vs* 25.13 ± 3.23, *p* < 0.001; Figure 5B), whereas OA treatment significantly reduced infarct size. The ELISA results show that the serum levels of cTnI, cTnT, CK-MB, LDH, AST are higher after MI/RI when compared with the Sham group (0.28 ± 0.02 *vs* 1.75 ± 0.16, 0.48 ± 0.05 *vs* 2.58 ± 0.15, 215.10 ± 27.35 *vs* 1,088.02 ± 76.45, 1,314.00 ± 91.39 *vs* 2422.00 ± 166.50, and 111.50 ± 15.31 *vs* 326.50 ± 20.17, respectively, *p* < 0.001; Figure 5C–G). However, the levels of myocardial enzymes after OA treatment were lower than those in the MI/RI group.

### OA Treatment Ameliorates Cardiac Function Against MI/RI

**FIGURE 6:**
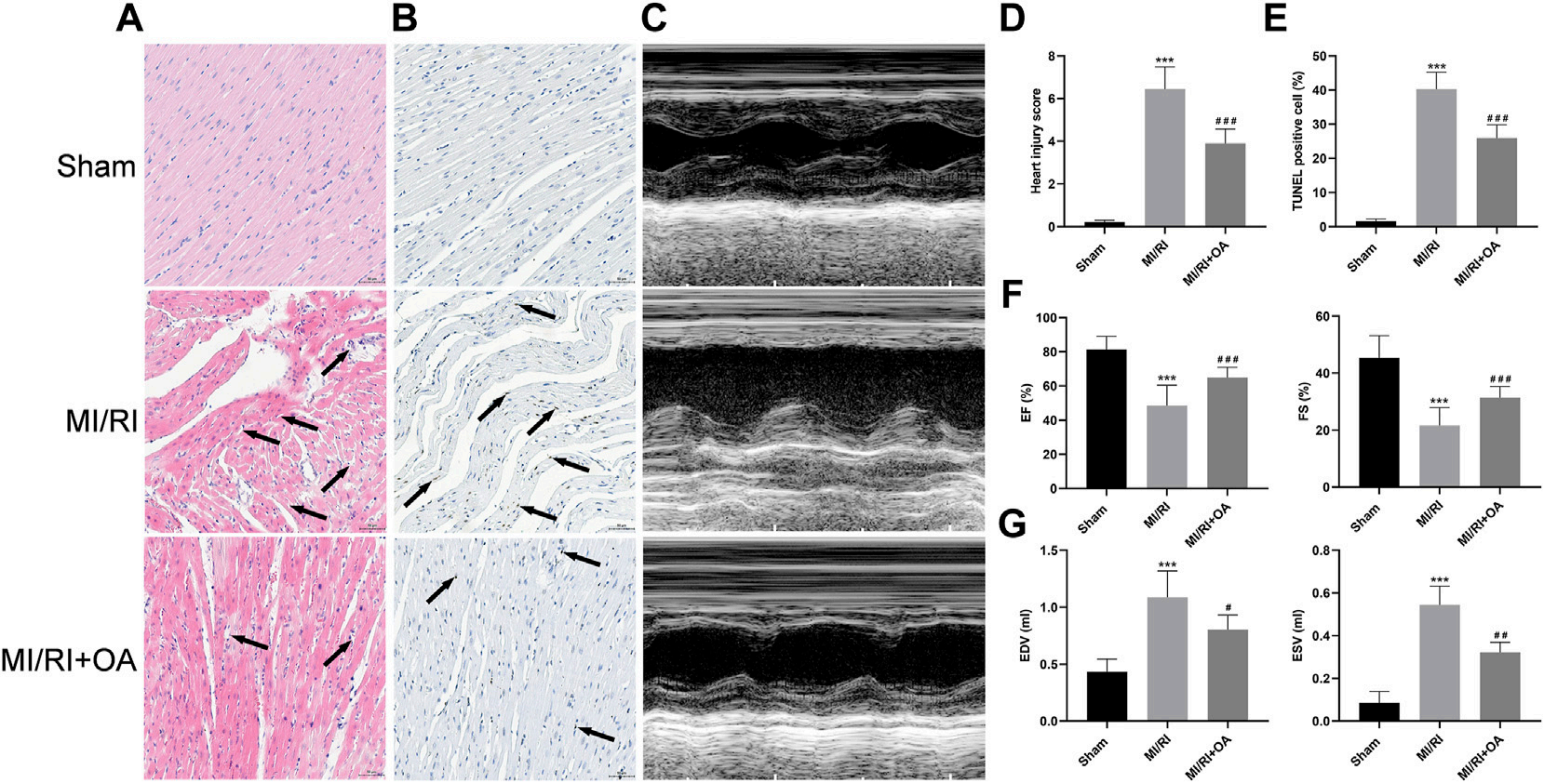
OA treatment ameliorates cardiac function against MI/RI **(A,D)** Representative H&E staining images and quantitative analysis for heart tissues in each group (scale bar = 50 μm). Arrows indicate edema and inflammation cell infiltration **(B,E)** Representative TUNEL staining images and quantitative analysis for heart tissues in each group (scale bar = 50 μm). Arrows indicate representative apoptotic positive cells **(C,F,G)** Representative Echocardiography images and quantitative analysis of EF, FS, EDV, ESV for heart tissues in each group. Data are presented as the mean ± SD. ^∗^ *p* < 0.05, compared with the Sham group; ^#^ *p* < 0.05, compared with the MI/RI group. MI/RI: myocardial ischemia/reperfusion injury; OA: Oroxin A; H&E: hematoxylin and eosin; TUNEL: Terminal-deoxynucleotidyl transferase-mediated nick end labeling; EF: ejection fraction; FS: fractional shortening; EDV: end diastolic volume; ESV: end systolic volume.

H&E staining was used to observe the morphology of myocardial tissues (Figure 6A). The results show that myocardial structural disorder, edema and inflammatory cells infiltration are more severe in the MI/RI group than that in the Sham group (0.22 ± 0.09 *vs* 6.45 ± 1.04, *p* < 0.001; Figure 6D), which improved after OA treatment. Myocardial apoptosis was detected by TUNEL staining (Figure 6B). The results show that apoptosis cells are markedly higher in the MI/RI group when compared with the Sham group (1.69 ± 0.64 *vs* 40.27 ± 4.95, *p* < 0.001; Figure 6E), which were reduced after OA treatment. Echocardiography was performed to evaluate cardiac function (Figure 6C). EF and FS significantly decreased after MI/RI when compared with the Sham group (81.38 ± 7.64 *vs* 48.56 ± 11.80, and 45.40 ± 7.77 *vs* 21.62 ± 6.35, respectively, *p* < 0.001; Figure 6F), while OA treatment greatly increased EF and FS. EDV and ESV were higher in the MI/RI group than that in the Sham group (0.44 ± 0.11 *vs* 1.09 ± 0.23, and 0.09 ± 0.05 *vs* 0.55 ± 0.09, respectively, *p* < 0.001; Figure 6G), while OA treatment greatly reduced EDV and ESV after MI/RI.

### OA Treatment Ameliorates MI/RI Induced Pyroptosis *in vivo*

**FIGURE 7:**
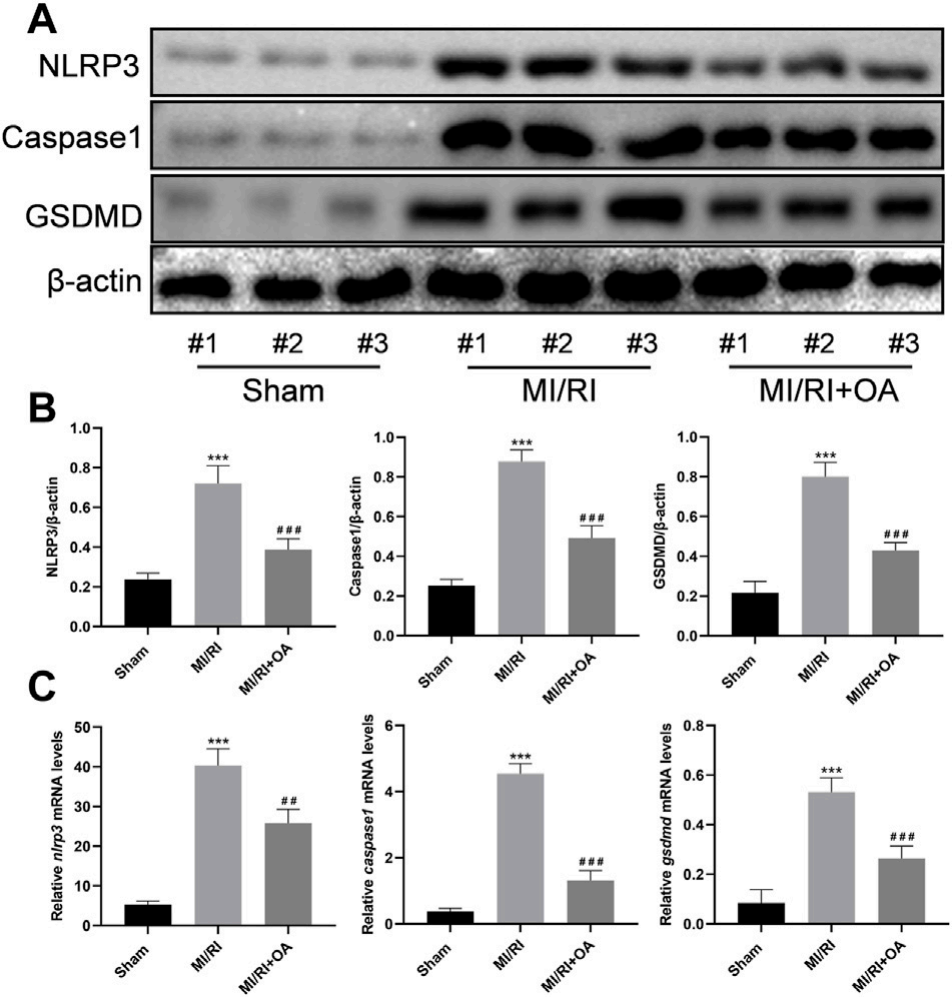
OA treatment ameliorates MI/RI induced pyroptosis *in vivo* **(A,B)** WB and quantitative analysis for NLRP3, Caspase1, and GSDMD expression levels in the heart tissues **(C)** RT-qPCR data showing that the transcripts levels for *nlrp3*, *caspase1*, and *gsdmd* in the heart tissues. Data are presented as the mean ± SD. ^∗^ *p* < 0.001, compared with the Sham group; ^**#**^ *p* < 0.001, compared with the MI/RI group. MI/RI: myocardial ischemia/reperfusion injury; WB: Western Blot; RT-qPCR: reverse-transcription quantitative PCR; OA: Oroxin A.

The expressions of pyroptosis related proteins were detected using WB (Figure 7A). Compared with the Sham group, the NLRP3, Caspase1, and GSDMD levels were significantly higher in the MI/RI group (0.24 ± 0.03 *vs* 0.72 ± 0.09, 0.25 ± 0.03 *vs* 0.88 ± 0.06, and 0.22 ± 0.06 *vs* 0.80 ± 0.07, respectively, *p* < 0.001; Figure 7B). However, OA treatment markedly decreased the above-mentioned proteins’ expressions. The RT-qPCR results show that the *nlrp3*, *caspase1*, and *gsdmd* mRNA levels are significantly higher in the MI/RI group when compared with the Sham group (5.28 ± 0.91 *vs* 40.34 ± 4.20, 0.37 ± 0.10 *vs* 4.54 ± 0.30, and 0.08 ± 0.05 *vs* 0.53 ± 0.06, respectively, *p* < 0.001; Figure 7C). However, OA treatment markedly decreased these mRNA expressions.

### OA Treatment Attenuates MI/RI Induced Inflammation *in vivo*

**FIGURE 8:**
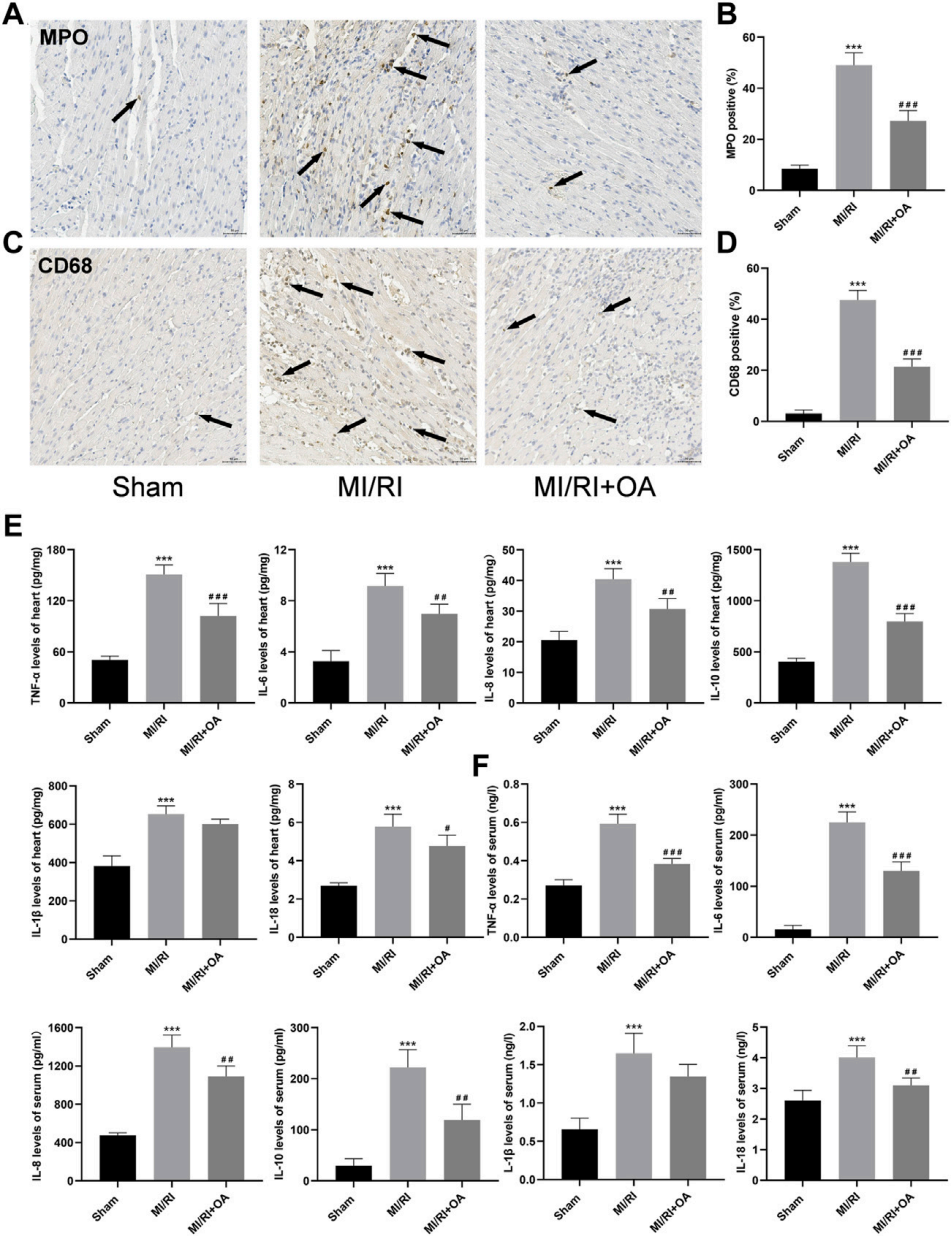
OA treatment attenuates MI/RI induced inflammation *in vivo* **(A–D)** Representative IHC images and quantitative analysis for MPO and CD68 in each group (scale bar = 50 μm). Arrows indicate representative neutrophil and macrophagocyte infiltrations **(E,F)** ELISA results showing that levels of TNF-α, IL-6, IL-8, IL-10, IL-1β and IL-18 in the heart and serum in each group. Data are presented as the mean ± SD. ^∗∗∗^ *p* < 0.001, compared with the Sham group; ^#^ *p* < 0.05, ^##^ *p* < 0.01, ^###^ *p* < 0.001, compared with the MI/RI group. MI/RI: myocardial ischemia/reperfusion injury; OA: Oroxin A; MPO: myeloperoxidase; ELISA: enzyme-linked immunosorbent assay.

To further determine the effects of OA on MI/RI induced inflammation *in vivo*, IHC and ELISA assays were performed. As shown in Figure 8A–D, the number of neutrophils (MPO for biomarker of the neutrophil) and macrophage (CD68 for biomarker of the macrophages) infiltrations into the injured myocardium was significantly higher in the MI/RI group when compared to those in the Sham group (8.53 ± 1.34 *vs* 49.08 ± 4.86 and 3.09 ± 1.37 *vs* 47.58 ± 3.73, respectively, *p* < 0.001). However, OA treatment decreased the levels of inflammatory infiltrations. The ELISA results show that the levels of TNF-α, IL-6, IL-8, IL-10, IL-1β, and IL-18 in heart tissues and serum are higher after MI/RI when compared with the Sham group (50.69 ± 4.26 *vs* 151.00 ± 10.98, 3.27 ± 0.83 *vs* 9.16 ± 0.99, 20.58 ± 2.87 *vs* 40.44 ± 3.47, 405.10 ± 31.38 *vs* 1,379.00 ± 86.54, 381.30 ± 53.72 *vs* 652.30 ± 43.13, and 2.69 ± 0.15 *vs* 5.78 ± 0.65 for the heart, respectively, *p* < 0.001; 0.27 ± 0.03 *vs* 0.59 ± 0.05, 15.71 ± 7.61 *vs* 224.80 ± 20.92, 475.40 ± 26.83 *vs* 1,396.00 ± 126.70, 28.89 ± 13.55 *vs* 222.30 ± 34.77, 0.65 ± 0.15 *vs* 1.65 ± 0.26, 2.61 ± 0.34 *vs* 4.01 ± 0.38 for the serum, respectively, *p* < 0.001; Figure 4E,F). Conversely, the levels of the above-mentioned inflammatory cytokines were markedly reduced after OA treatment.

## Discussion

The available evidence shows that inflammation and pyroptosis play a key role in the mechanism of MI/RI (Ou et al., 2022; Shen et al., 2022). The regulation of inflammatory response and the inhibition of pyroptosis may protect the myocardial from MI/RI injury. Through the establishment of an MI/RI model *in vivo* and *in vitro*, a natural compound named OA was selected using a HTS strategy, providing a novel alternative for the treatment of MI/RI.

As a flavonoid glycoside, OA has been shown to have antioxidant, anti-inflammatory, anti-tumor and immunomodulatory functions (Qiu et al., 2013; He et al., 2016; Pei et al., 2022). An OILK toxicity study showed that the maximum tolerated dose of Oroxin in mice was more than 500 mg/kg (Sun et al., 2017). Baicalein, as an OA aglycone, was also safe and well-tolerated in phase I clinical trials of a single oral dose of 100–2800 mg in healthy subjects (Li et al., 2014; Sun et al., 2018). These data indicate that OA has low toxicity and few side effects. Therefore, OA is considered a good candidate drug with low toxicity.

During the myocardial reperfusion phase, cardiomyocytes express various inflammatory cytokines, including chemokines and pro-inflammatory cytokines (IL-6, IL-8, TNF-α), that contribute to neutrophil and macrophage infiltration of the myocardium (Fan et al., 2019; Yan et al., 2021). As an anti-inflammatory cytokine, IL-10 limits the progression of myocardial inflammation (Roumeliotis et al., 2021). The IHC detection showed that the infiltration of MPO and CD68 positive cells in the myocardium of OA treated rats with reperfusion injury was significantly lower. The ELISA results show that pro-inflammatory and anti-inflammatory cytokines significantly decreased in the OA group. In the *in vitro* experiment, the inflammatory response in the cell supernatant after OA treatment was also significantly inhibited.

Pyroptosis is a type of programmed cell death mediated by the gasdermin family, in which the NLRP3 inflammasome plays an important role (Shi et al., 2021; Shen et al., 2022). Substantial evidence suggests that activation of the NLRP3 inflammasome in the ischemic heart can exacerbate the myocardial injury, dysfunction, and heart failure through dysregulation of apoptosis, autophagy, oxidative stress, and cardiac inflammation (Shen et al., 2022). Pharmacological or genetic inhibition of NLRP3 and its downstream Caspase1 can effectively alleviate MI/RI by inhibiting the cleavage of IL-1β and IL-18 precursors. IL-18 and IL-1β are two main downstream products of NLRP3 (Mastrocola et al., 2016). Notably, the activation of NLRP3 resulted in the activation of Caspase-1 and the production of IL-18, but not IL-1β release in cardiomyocytes (Woldbaek et al., 2003; Xiao et al., 2018). This study found that OA could significantly inhibit the expressions of pyroptosis-related proteins and mRNAs after MI/RI in rats. The content of IL-18 in myocardial tissue and serum was also significantly reduced, while the expression level of IL-1β was not affected by OA, which is consistent with previous studies (Zhao et al., 2021). Myocardial enzymes, such as cTnI, cTnT, CK-MB, LDH and AST produced in cardiomyocytes, can be released from damaged tissue into the blood circuit under pathological conditions (Zhu et al., 2021). This serum myocardial zymogram can be used as a reliable index to determine the integrity of the myocardial membrane and the degree of myocardial injury (Zhou et al., 2021). Combined with the observation of myocardial infarct size in rats, these results confirm the protective effect of OA on MI/RI.

Although this study attempted to enrich the experimental data, some limitations still exist. Firstly, H9c2 cells were selected in the *in vitro* experiment, which lack the morphological characteristics of primary cardiomyocytes and cannot accurately simulate cardiomyocytes. Secondly, in this study, we had only set a single dose (5 mg/kg) for the MIRI + OA group, although OA has a protective effect on MI/RI in animal experiments, its on myocardium has not yet been studied in a dose-dependent and time-dependent manner. Thirdly, given that OA can alleviate the occurrence and development of diabetes. In the future study, we will establish a diabetic rat model to further explore the effect of OA on MI/RI in dysmetabolic rat model. Finally, OA have anti-pyroptosis and anti-apoptotic properties, but its anti-pyroptosis effects in other molecules or pathways during MI/RI deserve further study.

## Conclusion

In conclusion, the results of this study suggest, for the first time, that OA can inhibit inflammation, pyroptosis and apoptosis in MI/RI, both *in vivo* and *in vitro*. The characteristics of OA provide a novel treatment strategy for MI/RI.

## Data Availability

The raw data supporting the conclusions of this article will be made available by the authors, without undue reservation.

## References

[B26] Dianita R. Jantan I. Amran A. Z. Jalil J. (2015). Protective effects of Labisia pumila var. alata on biochemical and histopathological alterations of cardiac muscle cells in isoproterenol-induced myocardial infarction rats. Molecules 20 (3), 4746–4763. 10.3390/molecules20034746 25786162 PMC6272229

[B1] Dinda B. SilSarma I. Dinda M. Rudrapaul P. (2015). Oroxylum Indicum (L.) Kurz, an Important Asian Traditional Medicine: From Traditional Uses to Scientific Data for its Commercial Exploitation. J. Ethnopharmacol. 161, 255–278. 10.1016/j.jep.2014.12.027 25543018

[B2] Fan Q. Tao R. Zhang H. Xie H. Lu L. Wang T. (2019). Dectin-1 Contributes to Myocardial Ischemia/Reperfusion Injury by Regulating Macrophage Polarization and Neutrophil Infiltration. Circulation 139 (5), 663–678. 10.1161/circulationaha.118.036044 30586706

[B3] Gu L. Sun M. Li R. Zhang X. Tao Y. Yuan Y. (2022). Didymin Suppresses Microglia Pyroptosis and Neuroinflammation through the Asc/Caspase-1/GSDMD Pathway Following Experimental Intracerebral Hemorrhage. Front. Immunol. 13, 810582. 10.3389/fimmu.2022.810582 35154128 PMC8828494

[B4] He J. Du L. Bao M. Zhang B. Qian H. Zhou Q. (2016). Oroxin A Inhibits Breast Cancer Cell Growth by Inducing Robust Endoplasmic Reticulum Stress and Senescence. Anticancer Drugs 27 (3), 204–215. 10.1097/cad.0000000000000318 26599214

[B5] Li D. Q. Zhao J. Li S. P. Zhang Q. W. (2014). Discovery of Xanthine Oxidase Inhibitors from a Complex Mixture Using an Online, Restricted-Access Material Coupled with Column-Switching Liquid Chromatography with a Diode-Array Detection System. Anal. Bioanal. Chem. 406 (7), 1975–1984. 10.1007/s00216-013-7612-8 24510210

[B6] Mastrocola R. Penna C. Tullio F. Femminò S. Nigro D. Chiazza F. (2016). Pharmacological Inhibition of NLRP3 Inflammasome Attenuates Myocardial Ischemia/Reperfusion Injury by Activation of RISK and Mitochondrial Pathways. Oxid. Med. Cell. Longev. 2016, 5271251. 10.1155/2016/5271251 28053692 PMC5178375

[B7] Milanlouei S. Menichetti G. Li Y. Loscalzo J. Willett W. C. Barabási A. L. (2020). A Systematic Comprehensive Longitudinal Evaluation of Dietary Factors Associated with Acute Myocardial Infarction and Fatal Coronary Heart Disease. Nat. Commun. 11 (1), 6074. 10.1038/s41467-020-19888-2 33247093 PMC7699643

[B8] Moya E. L. J. Vandenhaute E. Rizzi E. Boucau M. C. Hachani J. Maubon N. (2021). Miniaturization and Automation of a Human *In Vitro* Blood-Brain Barrier Model for the High-Throughput Screening of Compounds in the Early Stage of Drug Discovery. Pharmaceutics 13 (6), 892. 10.3390/pharmaceutics13060892 34208550 PMC8233835

[B9] Ou D. Ni D. Li R. Jiang X. Chen X. Li H. (2022). Galectin-1 Alleviates Myocardial Ischemia-Reperfusion Injury by Reducing the Inflammation and Apoptosis of Cardiomyocytes. Exp. Ther. Med. 23 (2), 143. 10.3892/etm.2021.11066 35069824 PMC8756402

[B10] Pei T. Yan M. Li T. Li X. Yin Y. Cui M. (2022). Characterization of UDP-Glycosyltransferase Family Members Reveals How Major Flavonoid Glycoside Accumulates in the Roots of Scutellaria Baicalensis. BMC Genomics 23 (1), 169. 10.1186/s12864-022-08391-1 35232374 PMC8888134

[B11] Qiu J. Wang D. Zhang Y. Dong J. Wang J. Niu X. (2013). Molecular Modeling Reveals the Novel Inhibition Mechanism and Binding Mode of Three Natural Compounds to Staphylococcal α-Hemolysin. PLoS One 8 (11), e80197. 10.1371/journal.pone.0080197 24312202 PMC3842302

[B12] Rai D. Aswatha Ram H. N. Neeraj Patel K. Babu U. V. Sharath Kumar L. M. Kannan R. (2021). *In Vitro* immuno-stimulatory and Anticancer Activities of Oroxylum Indicum (L.) Kurz.: An Evidence for Substitution of Aerial Parts for Conservation. J. Ayurveda Integr. Med. 13 (2), 100523. 10.1016/j.jaim.2021.09.001 34823972 PMC8728068

[B13] Roumeliotis S. Veljkovic A. Georgianos P. I. Lazarevic G. Perisic Z. Hadzi-Djokic J. (2021). Association between Biomarkers of Oxidative Stress and Inflammation with Cardiac Necrosis and Heart Failure in Non-ST Segment Elevation Myocardial Infarction Patients and Various Degrees of Kidney Function. Oxid. Med. Cell. Longev. 2021, 3090120. 10.1155/2021/3090120 34760045 PMC8575633

[B14] Seefeldt J. M. Lassen T. R. Hjortbak M. V. Jespersen N. R. Kvist F. Hansen J. (2021). Cardioprotective Effects of Empagliflozin after Ischemia and Reperfusion in Rats. Sci. Rep. 11 (1), 9544. 10.1038/s41598-021-89149-9 33953281 PMC8100147

[B15] Seo E. H. Song G. Y. Namgung J. H. Oh C. S. Lee S. H. Kim S. H. (2018). Receptor for Activated C Kinase 1 in Rats with Ischemia-Reperfusion Injury: Intravenous Versus Inhalation Anaesthetic Agents. Int. J. Med. Sci. 15 (4), 352–358. 10.7150/ijms.22591 29511370 PMC5835705

[B16] Shen S. Wang Z. Sun H. Ma L. (2022). Role of NLRP3 Inflammasome in Myocardial Ischemia-Reperfusion Injury and Ventricular Remodeling. Med. Sci. Monit. 27, e934255. 10.12659/msm.934255 PMC8790935 35042840

[B17] Shi H. Gao Y. Dong Z. Yang J. Gao R. Li X. (2021). GSDMD-Mediated Cardiomyocyte Pyroptosis Promotes Myocardial I/R Injury. Circ. Res. 129 (3), 383–396. 10.1161/circresaha.120.318629 34015941 PMC8291144

[B18] Sun W. Sang Y. Zhang B. Yu X. Xu Q. Xiu Z. (2017). Synergistic Effects of Acarbose and an Oroxylum Indicum Seed Extract in Streptozotocin and High-Fat-Diet Induced Prediabetic Mice. Biomed. Pharmacother. 87, 160–170. 10.1016/j.biopha.2016.12.096 28056420

[B19] Sun W. Zhang B. Yu X. Zhuang C. Li X. Sun J. (2018). Oroxin A from Oroxylum Indicum Prevents the Progression from Prediabetes to Diabetes in Streptozotocin and High-Fat Diet Induced Mice. Phytomedicine 38, 24–34. 10.1016/j.phymed.2017.10.003 29425652

[B20] Woldbaek P. R. Tønnessen T. Henriksen U. L. Florholmen G. Lunde P. K. Lyberg T. (2003). Increased Cardiac IL-18 mRNA, pro-IL-18 and Plasma IL-18 after Myocardial Infarction in the Mouse; a Potential Role in Cardiac Dysfunction. Cardiovasc Res. 59 (1), 122–131. 10.1016/s0008-6363(03)00339-0 12829183

[B21] Xiao H. Li H. Wang J. J. Zhang J. S. Shen J. An X. B. (2018). IL-18 Cleavage Triggers Cardiac Inflammation and Fibrosis upon β-Adrenergic Insult. Eur. Heart J. 39 (1), 60–69. 10.1093/eurheartj/ehx261 28549109

[B22] Yan M. Yang Y. Zhou Y. Yu C. Li R. Gong W. (2021). Interleukin-7 Aggravates Myocardial Ischaemia/Reperfusion Injury by Regulating Macrophage Infiltration and Polarization. J. Cell. Mol. Med. 25 (21), 9939–9952. 10.1111/jcmm.16335 34581005 PMC8572772

[B23] Yu P. Zhang X. Liu N. Tang L. Peng C. Chen X. (2021). Pyroptosis: Mechanisms and Diseases. Signal Transduct. Target Ther. 6 (1), 128. 10.1038/s41392-021-00507-5 33776057 PMC8005494

[B24] Zhao G. Zhang H. Zhu S. Wang S. Zhu K. Zhao Y. (2021). Interleukin-18 Accelerates Cardiac Inflammation and Dysfunction during Ischemia/Reperfusion Injury by Transcriptional Activation of CXCL16. Cell. Signal 87, 110141. 10.1016/j.cellsig.2021.110141 34487815

[B25] Zhou J. Peng F. Cao X. Xie X. Chen D. Yang L. (2021). Risk Compounds, Preclinical Toxicity Evaluation, and Potential Mechanisms of Chinese Materia Medica-Induced Cardiotoxicity. Front. Pharmacol. 12, 578796. 10.3389/fphar.2021.578796 33867974 PMC8044783

